# Screening for tylosin and other antimicrobial residues in fresh and fermented (nono) cow milk in Delta state, South-South, Nigeria

**DOI:** 10.14202/vetworld.2020.458-464

**Published:** 2020-03-12

**Authors:** Onwumere-Idolor Onyinye Stella, Ekene Vivienne Ezenduka, Nwanta John Anaelom

**Affiliations:** 1 Public Health specialty, University of Nigeria, Study Center, College of Veterinary Surgeons, Nigeria; 2 Department of Veterinary Public Health and Preventive Medicine, University of Nigeria, Nsukka, Nigeria

**Keywords:** antimicrobials, dairy, milk, residue, tylosin

## Abstract

**Background and Aim:**

Milk is a food that contains almost all the essential nutrients needed for growth and maintenance for both young and old animals and man. Since quite nutritious and in high demand, dairy products should be free of both chemical and biological contaminants. Unfortunately, antibiotics used in the treatment of infectious diseases in dairy cattle are often seen in their products if withdrawal periods of those drugs are not observed before milking. This study aimed to detect the presence of antibiotics and the level of tylosin in fresh and fermented (nono) milk from cows in Delta state, Nigeria.

**Materials and Methods:**

Two hundred and five samples comprising 126 fresh milk and 79 fermented milk (nono) were sampled from Kwale, Ozoro, and Oleh representing one senatorial district of Delta state, South-South Nigeria. They were screened for the presence of tylosin and other antimicrobial residues using four-plate test and tylosin was confirmed with high-performance liquid chromatography.

**Results:**

Antibiotic residues were obtained in 76% and 85% of fresh milk and nono, respectively. Tylosin residue was also detected in 24% fresh milk and 11% nono samples at mean concentrations of 14.64±0.69 µg/l and 7.97±0.23 µg/l, respectively. The mean concentrations of tylosin from both milk types were below the recommended maximum residue limit (MRL) of 50 µg/l in dairy.

**Conclusion:**

High prevalence of antimicrobial residues in fresh milk and nono shows that consumers in the study area are predisposed to health hazards due to the presence of residues of different antibiotics in fresh milk and nono. Although tylosin was confirmed in both milk products, the mean concentrations were below the MRL. However, it is still very vital to resort to the observance of withdrawal periods and avoid gross misuse of antimicrobials. It is also necessary to emphasize the need for effective prevention of infectious diseases and most importantly create awareness and establish a national antibiotic residue monitoring program in Nigeria.

## Introduction

Milk is one of the most balanced food compositions which provide protein, calcium, fats, mineral potassium, phosphorus, vitamins, lactose, and amino acids for growth, maintenance, and development of body tissues. Therefore, it is an essential part of a daily diet for all ages: Children and the elderly [1]. Food for human consumption should be free of all types of compounds of adverse effects on human health. These harmful compounds are not restricted to microorganisms and biological factors and toxins excreted from them, but all types of chemicals that directly or indirectly contaminate foodstuff are also highly important. Unlike microbial infections and poisoning, complications of chemical poisonings often take effect in the long term.

In Nigeria, dairy industry is an important component of agribusiness sector of the economy with great economic, nutritional, and social endeavor [2], and as the largest milk producer in West Africa, Nigeria has the potential of being a major milk producer in Africa with total annual demand estimated at 1.45 billion liters [3]. Most dairy cattle in Nigeria are reared by the Fulani herdsmen, who most likely engage in indiscriminate use of veterinary drugs for prophylactic and therapeutic purposes in their cattle [4]. This is largely due to the nomadic system of cattle rearing where cattle are moved through the bush and road paths to different parts of the country, with limited access to veterinary clinics and veterinarians. They buy these drugs which are readily available over the counter and administer to the animals themselves with neither prescription nor correct dosage [5].

Antimicrobials are used in dairy cattle production primarily to treat/prevent diseases, increase milk production, or improve feed efficiency by administering at low doses for extended periods [6]. However, widespread use could lead to persistent residues in edible parts such as muscle (meat), organs, egg, and milk obtained from treated animals [7]. These residues may include the non-altered parent residue as well as metabolites and/or conjugates [8]. The presence of these residues is usually attributed to non-observance of withdrawal periods before slaughter and milking, which is mainly due to undesirable practices such as unregulated and indiscriminate use of drugs and lack of awareness of the proper use of these antibiotics [9,10]. There is also the undocumented claim that due to the lack of refrigeration, antimicrobials are added to fresh milk to avoid the activities of spoilage bacteria. Fresh milk and milk products are important products of the Fulani agro-pastoral and even other smallholders peri-urban system of Nigeria [11]. They are produced and processed by traditional herdsmen and milkmaids for local consumption.

Antibiotic residues in milk and products are of great concern to dairy farmers, milk processors, consumers, and regulatory agencies. To ensure food safety for consumers, several regulatory authorities around the world, including the European Food Safety Agency, Food and Drug Administration, and USA Codex Alimentarius, established tolerance (safe) levels of antibiotic residues in milk [12]. Exceeding tolerance levels not only present potential health risks to the consumer but also interfere with fermentation processes through the inhibition of starter culture during milk processing for the production of cheese and yogurt [13]. The concern of drug residues in milk to human health is due to their association with allergies and life-threatening hypersensitive reactions [14,15], development of resistant strains of microorganisms [16], and carcinogenic and mutagenic effects [17]. Different methods were developed to detect antibiotics in milk, screening and chromatographic techniques. Microbiological methods are basically used for screening, although recommended as the conventional and official method used due to its high throughput and simple procedure, it lacks specificity and can at most, produces a semi-quantitative result [18]. Chromatographic methods were, therefore, developed to confirm the presence and quantitate the concentration of incriminating antibiotics [19].

Several studies have reported the presence of antibiotic residues in different foods of animal origin [4,20,21]. However, limited information is available on the occurrence of tylosin residue in bovine milk in Nigeria. In common with many other developing countries, the awareness of the extent of drug residues in foods of animal origin, especially milk has not been very well created to stakeholders. In effect, policy-makers remain unaware of the extent of the problem, suggesting that no quality assurance program is put in place to checkmate this issue.

This study aimed to detect the presence of antibiotics and the level of tylosin in fresh and fermented (nono) milk from cows in Delta state, Nigeria.

## Materials and Methods

### Ethical approval and informed consent

Informed consents of the herdsmen and management of milk collection centers in the three local government areas (LGAs) were obtained. Milk samples were collected from milk collection centers.

### Study area

**Figure-1:**
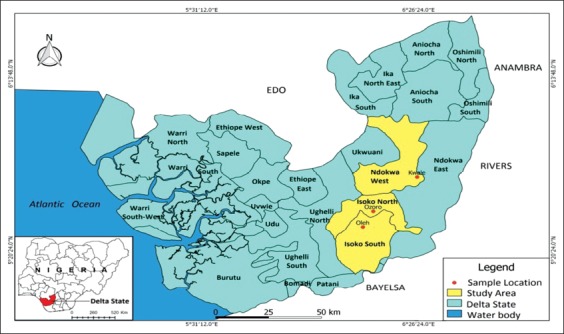
Map of the study area (Delta state) showing the sample collection points [Source: Wikipedia and made by the authors].

The study was carried out in three LGAs of Delta State, South-South Nigeria (Figure-1). The LGAs comprise Kwale representing Ndokwa West, Ozoro representing Isoko North, and Oleh representing Isoko South.

### Study design

A cross-sectional survey was carried out using both qualitative and quantitative approaches to determine the occurrence of antimicrobial residues using a four-plate test. Samples that were positive as macrolides were cleaned-up to obtain tylosin residue analytes using high-performance liquid chromatography (HPLC) to confirm the presence and determine the concentration of tylosin in fresh and fermented (nono) cow milk.

### Sample collection and transportation

The study used 20 ml each of 205 samples comprising 126 fresh milk and 79 fermented milk (nono) collected from Kwale, Ozoro, and Oleh Fulani milk-producing herds and dairy collection centers for a period of 8 weeks, using sterile universal sample bottles. The samples were immediately transported on ice cooler box to the Veterinary Public Health and Preventive Medicine laboratory of the University of Nigeria, Nsukka, for analysis. The samples were brought up to room temperature (27ºC) at the time of analysis.

### Screening for antimicrobial residue using four-plate test

Screening was done using the European four-plate test, a conventional microbiological test for the detection of antimicrobials residues in foods of animal origin.

#### Chemicals and reagents

Mueller-Hinton agar (Oxoid), hydrochloric acid (HCl), sodium hydroxide Na(OH)_4_, *Bacillus subtilis* (Merck Darmstadt, Germany, no. 64271), and *Micrococcus luteus* ATCC^®^ 10240 strain were used.

#### Procedure

**Figure-2:**
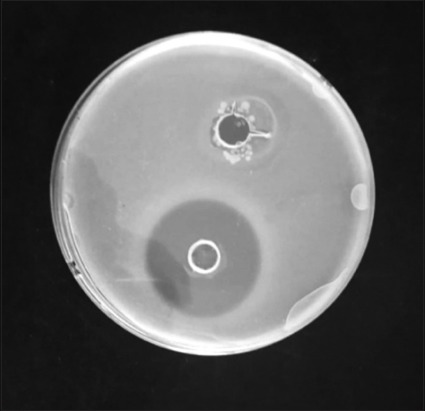
Four-plate test positive sample.

Four batches of Mueller-Hinton agar were prepared and adjusted to pH 6, 7.2, and 8 with dilute HCl as acid and Na(OH)_4_ as base. The first three plates with pH 6, 7.2, and 8 were seeded with *B. subtilis* which is ready to use suspension, the fourth plate, a pH 8 plate, was seeded with *M. luteus*. Two holes were bored on each plate. A 50 µl of the milk sample was inoculated in each hole and the other hole, 50 µl of distilled water as negative control. The plates were incubated at 30°C for 18-24 h, clear zone of inhibition with annular diameter of ≥2 mm indicates positive (Figure-2) for antimicrobial residues [22]. The plates seeded with *B. subtilis* a*t* pH 6.0 best detects β-lactams and tetracycline, at pH 7.2 best detects sulfonamides, and at pH 8.0 best detects aminoglycosides, while the plate seeded with *M. luteu*s at pH 8.0 best detects macrolides.

### Detection/confirmation of tylosin using HPLC

Detecting the presence of tylosin and its concentration from the positive samples of pH 8.0 plate seeded with *M. luteus* was done using HPLC, as described by Sokol *et al*. [23].

#### Reagents and chemicals

Tylosin tartrate standard was obtained from USP, 12601 Twin Brook Pkwy, Rockville, USA. Acetonitrile, methanol, and water HPLC grades were purchased from Scharlau Chemicals, Poland. Potassium dihydrogenorthophosphoric acid, methanol Analar grade, 5 ml plain sample bottle packs, 5 ml needle and syringes, 0.45 µm Millipore filters were purchased from ABIKTOL, Nigeria (distributors for Market Research Society, UK). C18 solid-phase extraction (SPE) cartridges Bond Elute C18 (500 mg/3 ml) were purchased from Supelco Park, Bellefonte, USA.

#### Tylosin standard solutions

Tylosin stock solution −1 mg/ml was prepared by dissolving 10 mg tylosin tartrate in 10 ml methanol and stored at −18°C. Working standard solutions for calibration curve were prepared by appropriate dilutions of the stock solution, using dilution factor. Fresh and nono milk tested and confirmed not to contain macrolide antibiotic residues (control) was used as sample blank for the preparation of matrix-matched calibration curve. For fortification, standard solutions were prepared by dissolving standard substance in methanol at concentrations of 40, 20, 10, 5, and 2.5 mg/ml.

#### Sample preparation

A 30 ml of fresh/nono milk was centrifuged for 10 min at 3000 rpm at 4°C to separate the fat layer. From the lower skim layer, 10 ml was transferred to another centrifuge tube and 20 ml acetonitrile was added. The solution was mixed and centrifuged for 10 min at 3000 rpm and 4°C. The extracted solvent layer was then decanted and diluted to 100 ml with water and applied to the SEP-Pak Vac C_18_ cartridge.

#### Clean-up procedure using SPE

The SPE cartridges Bond Elute C18 – 500 mg/3 ml was activated with 2 ml of methanol and 5 ml of water. The cartridge was washed with 20 ml of water at the flow of 1 drop/2 s and allowed to dry. The extracted sample solution was loaded and allowed to elute from the cartridge with 3 ml of methanol at the flow rate of 1 drop/s. The solution was filtered using 0.45 micromillipore syringe filter. The samples were manually injected into the chromatographic system.

#### Chromatographic condition

The chromatographic system used was an Agilent^®^ HPLC 1100 series system. Chromatographic analysis was performed with isocratic elution on Zorbax Eclipse XDB – C18 (150 mm×4.6 mm, 5 µm) analytical column at 30°C. The mobile phase composed of acetonitrile and water (90:10) at the flow rate of 1.00 ml/min. The volume injected was 20 µl at ultraviolet detection of 292 nm.

### Calculation of tylosin concentration from the standard curve as determined by HPLC

**Figure-3:**
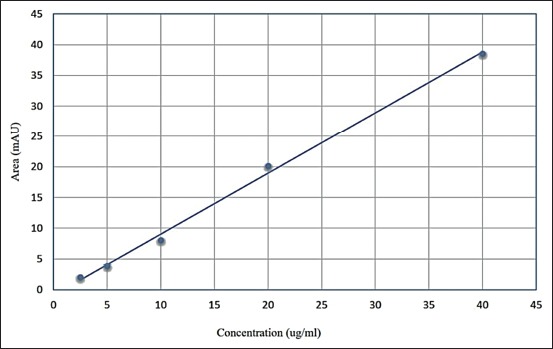
Tylosin analytical standard calibration curve.

**Figure-4:**
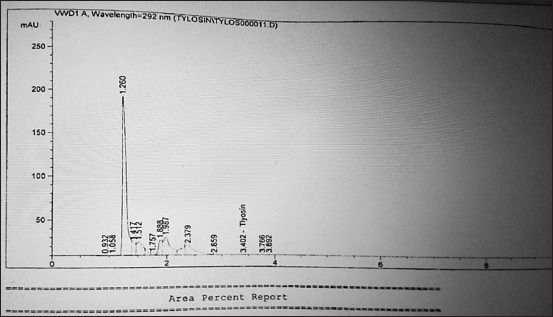
High-performance liquid chromatography chromatogram report of tylosin positive sample.

Figure-3 shows the results of the standard conc. and peak areas plotted as the standard curve produced a linear regression equation; y=0.9604x, where y=peak area (mAb) and x=conc. of tylosin, R2=correlation 0.9953, while Figure-4 shows positive tylosin sample.

### Data presentation and analysis

Data generated are presented in percentages in tables and charts. GraphPad Prism 5 statistical software version 5.02 for Windows (GraphPad Software, La Jolla, California, USA, www.graphpad.com) was used for the analysis where Chi-square was used to determine the association between the occurrence of residues and the location/milk type. t-test was used to determine if there is a difference between the mean concentrations of tylosin in fresh milk and nono, p˂0.05.

## Results

### Distribution of antimicrobial residues in fresh and nono milk samples in Kwale, Ozoro, and Oleh using four-plate test

A total of 205 dairy products were screened from three LGAs: Kwale, Ozoro, and Oleh of Delta state, comprising 126 fresh and 79 fermented (nono) milk, 163 (79.5%) were positive for antibiotic residues. Ninety-six (76%) out of 126 fresh milk and 67 (85%) out of 79 fermented milk (nono) samples were positive for antimicrobial residues.

For location distribution, 22 (88%) of 25, 25 (86%) of 29, and 20 (80%) of 25 nono samples from Ozoro, Kwale, and Oleh were positive for antimicrobial residues, respectively, while for fresh milk, 27 (54%) of 50, 45 (90%) of 50, and 24 (92%) of 26 were positive for Ozoro, Kwale, and Oleh, respectively (Table-1). There was a significant (Chi-square (2)=22.5; p<0.0001) association between the occurrence of antimicrobial residues in fresh milk and the location of sample collection, with Ozoro having the lowest occurrence.

**Table-1:** Location distribution of antimicrobial residues in fermented (nono) and fresh milk in Delta state.

Location	Sample type			

	Fermented milk (nono)		Fresh milk	
	
	No sampled	No positive (%)	No sampled	No positive (%)
Ozoro	25	22 (88)	50	27 (50)
Kwale	29	25 (86)	50	45 (90)
Oleh	25	20 (80)	26	24 (92)
Total	79	67 (84.8)	126	96 (76)

In the distribution of antimicrobial residues according to milk type, 96 (76%) of fresh milk and 67 (85%) of nono samples were positive for antimicrobial residue; however, the association between the occurrence of residue and the type of milk product was not significant (p=0.157).

### Tylosin residue in fresh milk and nono from the three local governments

Thirty (42%) out of the 71 presumptive macrolides containing fresh milk samples were confirmed tylosin, i.e., 24% of the whole fresh milk samples were positive for tylosin. Nine (23%) of the 39 presumptive macrolides containing nono samples were confirmed tylosin. The overall mean tylosin concentration was 14.64±0.6896 µg/l and 7.97±0.2265 µg/l for fresh milk and nono, respectively (Table-2), and the difference in the mean concentrations was statistically (t (17)=7.18; p<0.0001) significant.

**Table-2:** HPLC analysis of presumed macrolide from FPT for confirmation of tylosin in fermented (nono) and fresh milk in Delta state.

Location	Sample type					

	Fresh milk			Fermented milk (nono)		
	
	FPT +ve (M.L pH 8.0)	HPLC tylosin +ve	Mean conc. (µg/l)	FPT +ve (M.L pH 8.0)	HPLC tylosin +ve	Mean conc. (µg/l)
Ozoro	25	5	10.79	17	4	8.45
Kwale	30	21	15.38	10	3	7.62
Oleh	16	4	15.01	12	2	8.01
Total	71	30	14.64±0.6896	39	9	7.97±0.2265

HPLC=High-performance liquid chromatography, FPT=Frequent pattern tree

## Discussion

Milk is an important part of daily diet of all ages containing most balanced food compositions which provide essential nutrients for growth, maintenance, and development of body tissues. Being an important source of protein and other nutrients, it is in high demand, therefore should be free of contaminants and be safe for human consumption.

The study focused on determining the presence of antimicrobial residue in milk products (fresh milk and nono) that are ready to be consumed. The high prevalence of antibiotic residues in dairy products (fresh milk [76%] and fermented milk [85%]) in Delta state occurred at different pH levels of the frequent pattern tree (FPT), indicating the possible occurrence of beta-lactams, tetracyclines, aminoglycosides, and macrolides.

In Sweden, similar studies by Shitandi [10] and Shitandi and Sternesjo [24] have shown a range of the prevalence of 9-11% which is much lower than the result obtained from the present study. The disparity is not unexpected because Sweden, being a member of the European Union, has strong legislation and enforcement against the prudent use of antimicrobials in food-producing animals which is lacking in Nigeria. Although there seem to be laws guarding against the use of antimicrobials in food animals in Nigeria, it is hardly if ever enforced [21]. Studies carried out in Ethiopia [25] and Kenya [26] revealed the detection of antimicrobial residues in 8.5% raw milk and a range of 9-16%, respectively. The studies showed the low occurrence of antimicrobial residues probably because both countries have a more functional monitoring system of antimicrobials in slaughter animals. In Tanzania, antimicrobial residues were detected in 36% of marketed milk samples from milk supply chains in and around Mwanza and Dar es Salaam during 1999 and 2000 [27]. Mahmoudi *et al*. [28] recorded 29.16% in raw and pasteurized milk in Iran. In Ghana, a report showed that 35% of the raw milk marketed in two major cities, in Accra and Kumasi, were contaminated with antibiotics [29].

Regulations regarding veterinary drug use including withholding periods after antimicrobial use in food animals have been formulated, mainly to protect the public from the deleterious effects of the residues of these antimicrobials. Unfortunately, such regulations are rarely adhered to in some developing countries [29], especially Nigeria. This is buttressed by the work done by Olatoye *et al*. [4] in Southwest Nigeria which recorded overall prevalence of 42.6%, with 40.8%, 24.4%, and 62.3% in fresh milk, wara, and nono, respectively, and the high prevalence of 76% and 85% from fresh milk and nono, respectively, in this study. This implies that a large proportion of milk and milk products produced in the study area contains residues of one or more antibiotics. There is also a possibility of multi-antibiotic therapy for the treatment of infections, especially mastitis as most herders do not always have quick access to veterinarians; therefore, no sensitivity test done to narrow down on an antibiotic. The possibility of direct addition of antibiotics to bulk milk tanks for preservation is not ruled out. The degree of contamination of milk and dairy products with antimicrobial residues is dependent on the level of legislation and effectiveness of methods in tackling the indiscriminate use of antimicrobials in food animals in different countries [30]. The Ozoro samples had significantly lower occurrence of antimicrobial residues compared with Kwale and Oleh. Most of the samples collected from Ozoro were supplied to local fresh milk on producers from a ranch in a higher institution with an attached veterinarian. This shows that there is less indiscriminate use of antimicrobials in Ozoro, due to the intervention of a veterinarian. Apart from the assistance from the school veterinarian, Ozoro and Oleh, both have veterinary clinics run by qualified veterinarians which are sometimes patronized by herdsmen. This explains the lower number of positive samples compared with Kwale.

The high prevalence of antimicrobial residues recorded in this study indicates that consumers of locally produced fresh and nono milk are exposed to milk products with variable levels of antimicrobial residues, with the probability and risk of surpassing the acceptable daily intake for the various drugs.

The overall mean tylosin residue level in fresh milk (14.64 µg/l) obtained in this study is less than the Codex Alimentarius Commission maximum residue limits (MRLs) of 50 µg/l in milk and surprisingly lower than the reports by Ghidini *et al*. in Italy [31] (62.40±5.50) and Khaskheli *et al*. in Pakistan [32] (59.53 ug/l). The presence of antibiotic residue in foods of animal origin above the MRL is recognized worldwide as being deleterious to human health [33].

In general, the high prevalence of antimicrobial residues in fresh milk and nono samples in this study may be attributed to the fact that residue monitoring program has not been put in place by the government; neither has there been any effort to sensitize the public on the dangers associated with residues in animal products [9]. Widespread and unrestricted use of different antibiotics in food animals without adequate diagnosis, prescription, and supervision by veterinarians contribute greatly to the deposit of these residues of these drugs in dairy products. Non-observance of withdrawal period of a drug after treatment cannot be ruled out, the results clearly show that herders and farmers do not wait for the withdrawal periods of the drug before milking; hence, the likely presence of antimicrobial residues above the specific MRLs shown by the inhibition zones much above 2 mm in the FPT.

Antibiotic residues enter the milk supply chain at farm level. Therefore, producers must realize the factors that lead to antibiotic residues in milk and how these residues can be avoided. Furthermore, the milk testing program should become a component of the quality control process centered on the farm.

## Conclusion

More than half of the fresh and fermented milk sampled in this study contain antimicrobial residues. The possible detection of more than one antimicrobial in a sample is indicative of the use of more than one antimicrobial for treatment/prophylaxis. This is consequent on the easy availability of these antimicrobials over the counter and self-medication by the herdsmen, which means that the public is at risk of experiencing the health effects associated with consumption of such antimicrobial contaminated milk. The health risk associated with antimicrobial residues in milk and dairy products will continue to exist in Delta state and Nigeria as a whole until an effective residue monitoring program is legislated.

## Authors’ Contributions

EVE and OOS conceived the work, collected and analyzed the samples. EVE statistically analyzed the generated data. NJA and EVE drafted the manuscript. All the authors reviewed, edited, read, and approved the final manuscript.
